# Supplementary material for: Structural and catalytic diversity of coronavirus proofreading exoribonuclease
Source: Nat Commun. 2025 Dec 8;17:452. doi: 10.1038/s41467-025-67140-6 (PMC12800083; doi:10.1038/s41467-025-67140-6)
Supplement: Supplementary file 1 — Supplementary Information [file 41467_2025_67140_MOESM1_ESM.pdf]

Supplementary Information for

Structural and catalytic diversity of coronavirus proofreading exoribonuclease

Yu Li[1], Xiaocong Cao[2], Lauren M. Recker[1], Yang Yang[1,*], Chang Liu[2,*]

*Corresponding author. Email: yan9yang@iastate.edu (Y.Y.); cliu207@jhmi.edu (C.L.)

The PDF file includes:

Supplementary Figures 1–9

Supplementary Tables 1–2

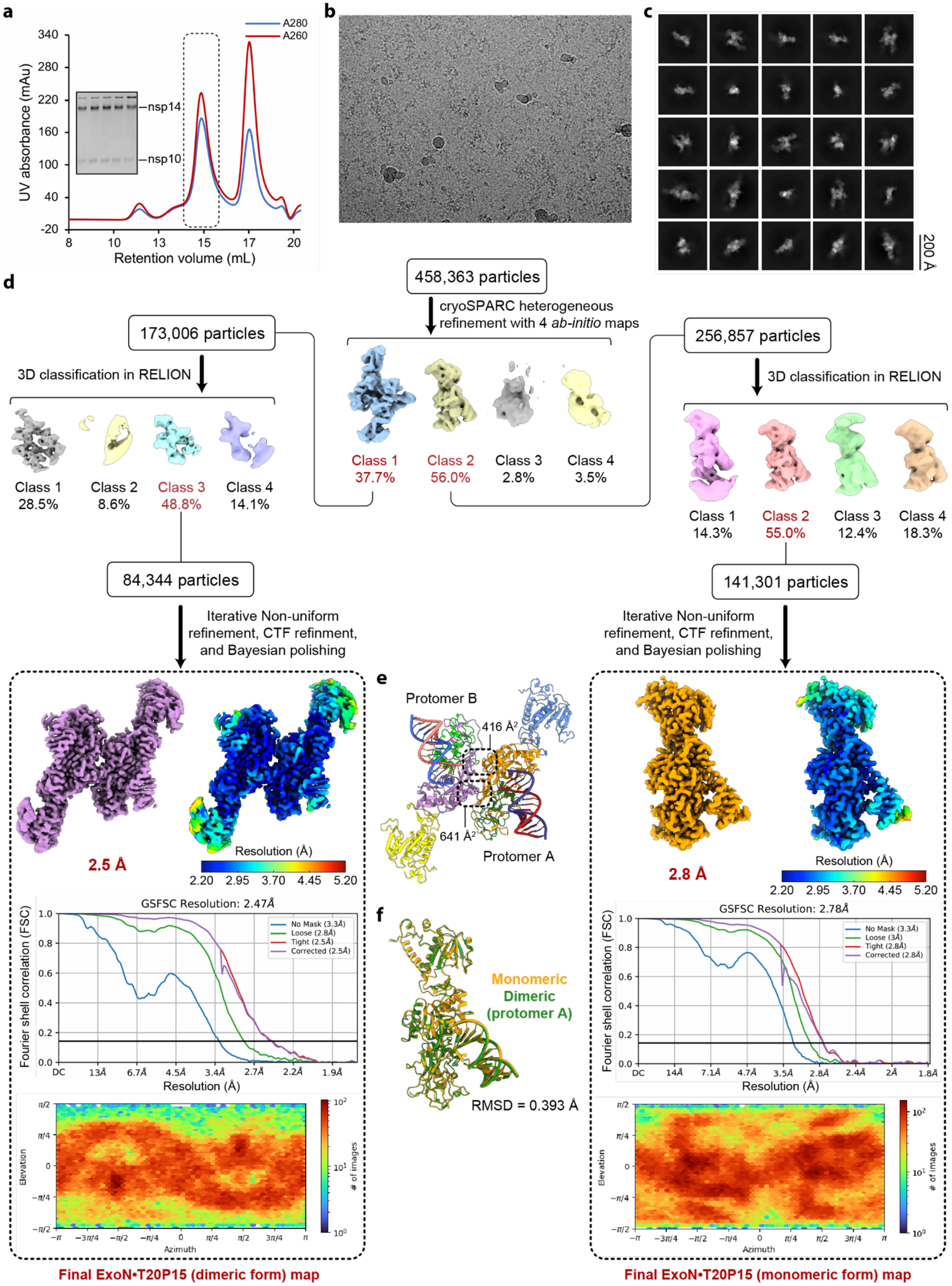
a
A280
A260
UV absorbance (mAu)
nsp14
nsp10
Retention volume (mL)
b
c
200 Å
d
458,363 particles
cryoSPARC heterogeneous refinement with 4 ab-initio maps
Class 1 37.7%
Class 2 56.0%
Class 3 2.8%
Class 4 3.5%
173,006 particles
3D classification in RELION
Class 1 28.5%
Class 2 8.6%
Class 3 48.8%
Class 4 14.1%
84,344 particles
Iterative Non-uniform refinement, CTF refinment, and Bayesian polishing
256,857 particles
3D classification in RELION
Class 1 14.3%
Class 2 55.0%
Class 3 12.4%
Class 4 18.3%
141,301 particles
Iterative Non-uniform refinement, CTF refinment, and Bayesian polishing
2.5 Å
Resolution (Å)
GSFSC Resolution: 2.47Å
Fourier shell correlation (FSC)
No Mask (3.3Å)
Loose (2.8Å)
Tight (2.5Å)
Corrected (2.5Å)
Resolution (Å)
Elevation
Azimuth
# of images
e
Protomer B
416 Å²
641 Å²
Protomer A
f
Monomeric
Dimeric (protomer A)
RMSD = 0.393 Å
2.8 Å
Resolution (Å)
GSFSC Resolution: 2.78Å
No Mask (3.3Å)
Loose (3Å)
Tight (2.8Å)
Corrected (2.8Å)
Final ExoN•T20P15 (dimeric form) map
Final ExoN•T20P15 (monomeric form) map

Supplementary Fig. 1: Single-particle cryo-EM analysis of MERS-CoV ExoN•T20P15 complex. (**a**) SEC purification of MERS-CoV ExoN•T20P15 complex. The protein compositions in the dotted-line boxed fractions were analyzed by SDS-PAGE and are shown in the embedded gel panel. (**b**) A representative micrograph from the ExoN•T20P15 complex cryo-EM dataset. (**c**) Representative 2D classes generated from the ExoN•T20P15 complex cryo-EM dataset. (**d**) Flow chart of cryo-EM image processing and map reconstruction for the ExoN•T20P15 complex. Heterogeneous refinement with four *ab-initio* reconstructed maps in cryoSPARC generated four major 3D classes, with two of them showing structural features corresponding to a dimeric and monomeric forms of ExoN, respectively. The particles from the two 3D classes were separately subjected to another round of 3D classification. The particles from the predominant 3D class, which shows the best map features, were selected for iterative non-uniform refinement, CTF refinement, and Bayesian polishing. The final cryo-EM map of the complex, local resolution illustrations, the accompanying half-map FSC plots, and the angular distribution plot of particles used in the final reconstruction are enclosed in the dashed box. (**e**) Overall structure of the dimeric form of MERS-CoV ExoN•T20P15 complex. (**f**) Superimposition of the monomeric form of the ExoN•T20P15 complex with protomer A from the dimeric form of this complex. Root-mean-square deviation (RMSD) of the superimposition is indicated. Source data are provided as a Source Data file.

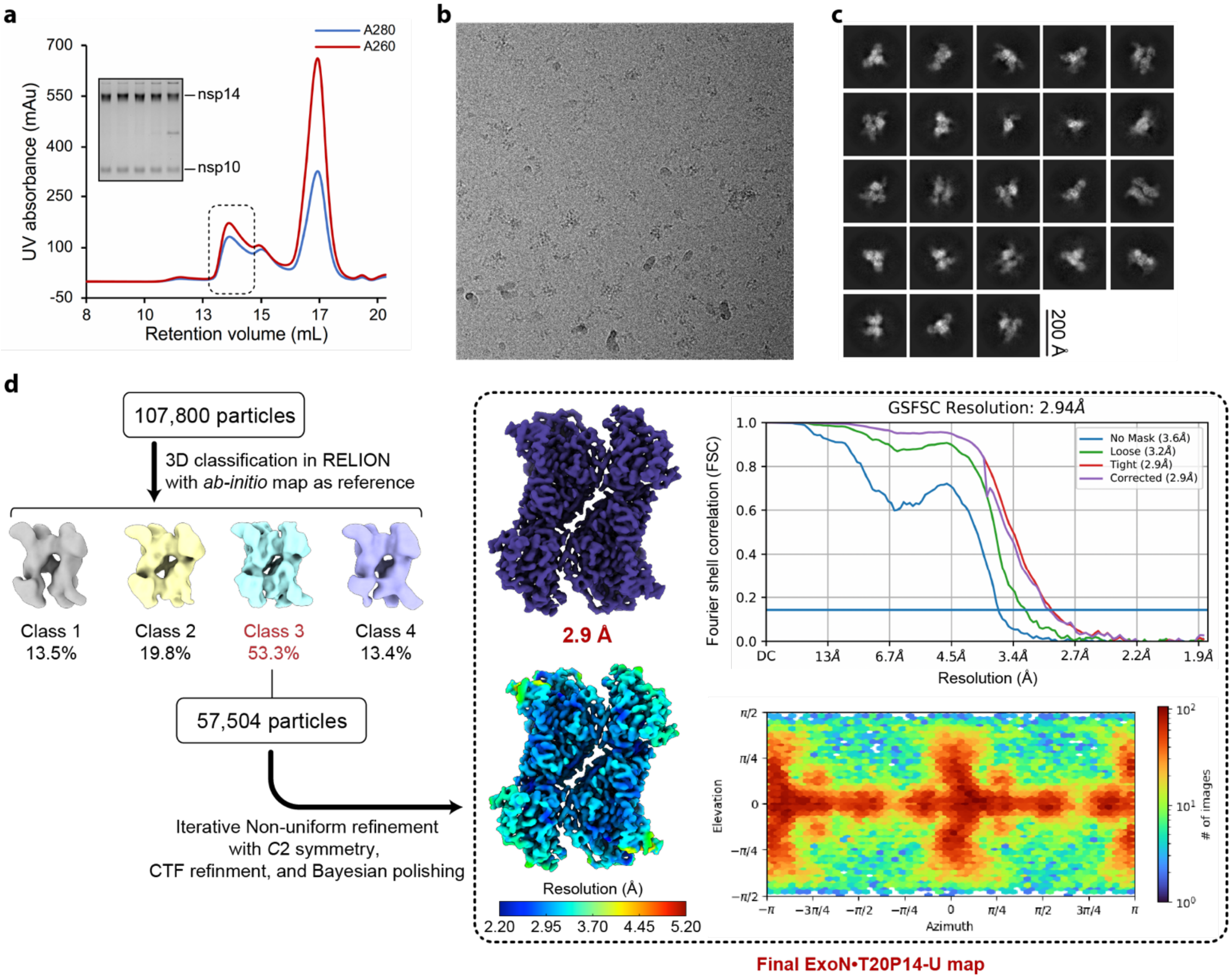

Supplementary Fig. 2: Single-particle cryo-EM analysis of MERS-CoV ExoN•T20P14-U complex. (**a**) SEC purification of MERS-CoV ExoN•T20P14-U complex. The protein compositions in the dotted-line boxed fractions were analyzed by SDS-PAGE and are shown in the embedded gel panel. (**b**) A representative micrograph from the ExoN•T20P14-U complex cryo-EM dataset. (**c**) Representative 2D classes generated from the ExoN•T20P14-U complex cryo-EM dataset. (**d**) Flow chart of cryo-EM image processing and map reconstruction for the ExoN•T20P14-U complex. Global 3D classification using an *ab-initio* reconstructed map as the reference generated four major 3D classes. The particles from the predominant 3D class, which shows the best map features, were selected for iterative non-uniform refinement with *C*2 symmetry, CTF refinement, and Bayesian polishing. The final cryo-EM map of the complex, the accompanying half-map FSC plots, local resolution illustrations, and the angular distribution plot of particles used in the final reconstruction are enclosed in the dashed box. Source data are provided as a Source Data file.

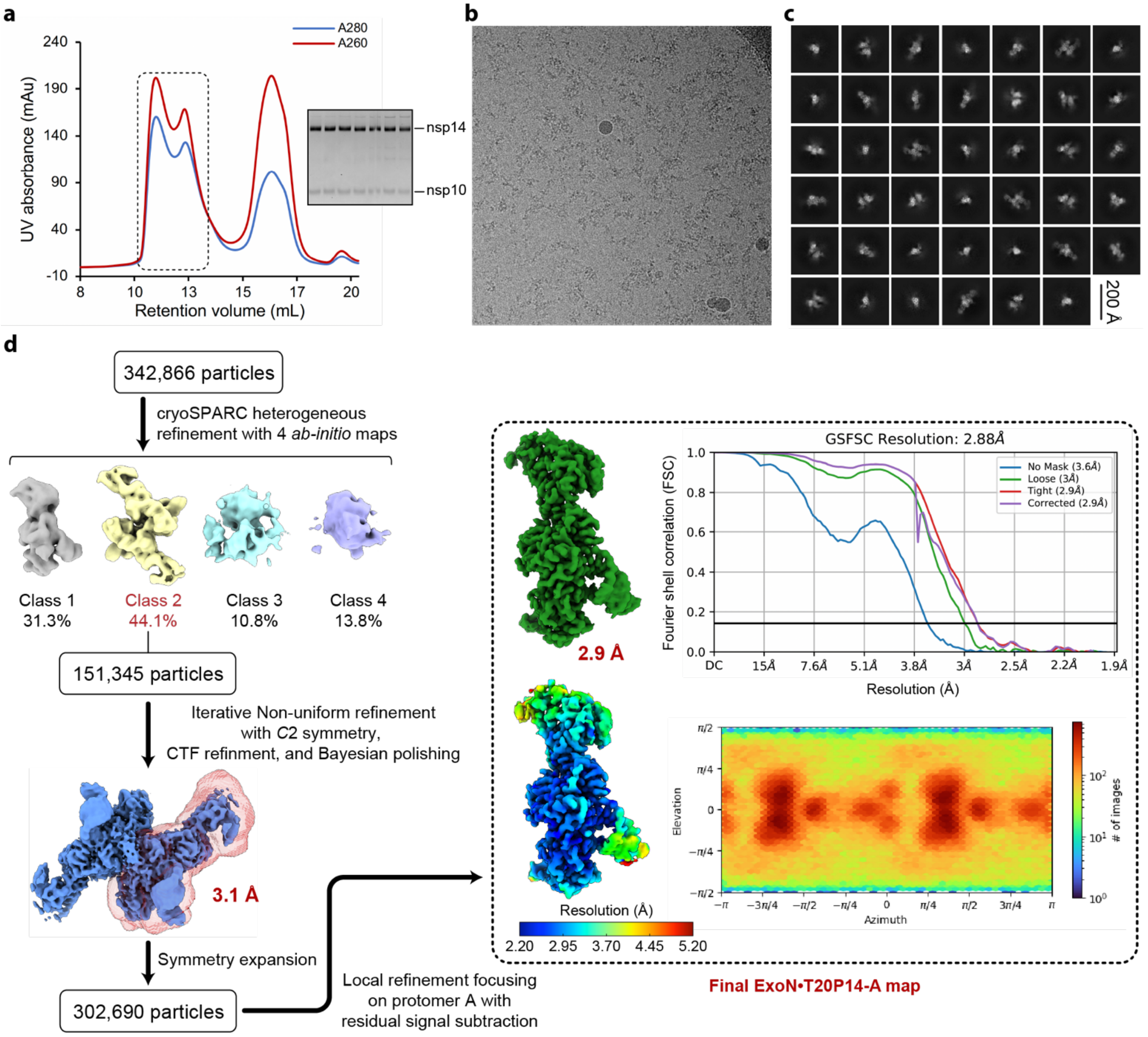

Supplementary Fig. 3: Single-particle cryo-EM analysis of MERS-CoV ExoN•T20P14-A complex. (**a**) SEC purification of MERS-CoV ExoN•T20P14-A complex. The protein compositions in the dotted-line boxed fractions were analyzed by SDS-PAGE and are shown in the embedded gel panel. (**b**) A representative micrograph from the ExoN•T20P14-A complex cryo-EM dataset. (**c**) Representative 2D classes generated from the ExoN•T20P14-A complex cryo-EM dataset. (**d**) Flow chart of cryo-EM image processing and map reconstruction for the ExoN•T20P14-A complex. Heterogeneous refinement with four *ab-initio* reconstructed maps in cryoSPARC generated four major 3D classes. The particles from the predominant 3D class, which shows the structural features corresponding to a dimeric form of ExoN•T20P14-A complex, were selected for iterative non-uniform refinement, CTF refinement, and Bayesian polishing. The final cryo-EM map of the complex, the accompanying half-map FSC plots, local resolution illustrations, and the angular distribution plot of particles used in the final reconstruction are enclosed in the dashed box. Source data are provided as a Source Data file.

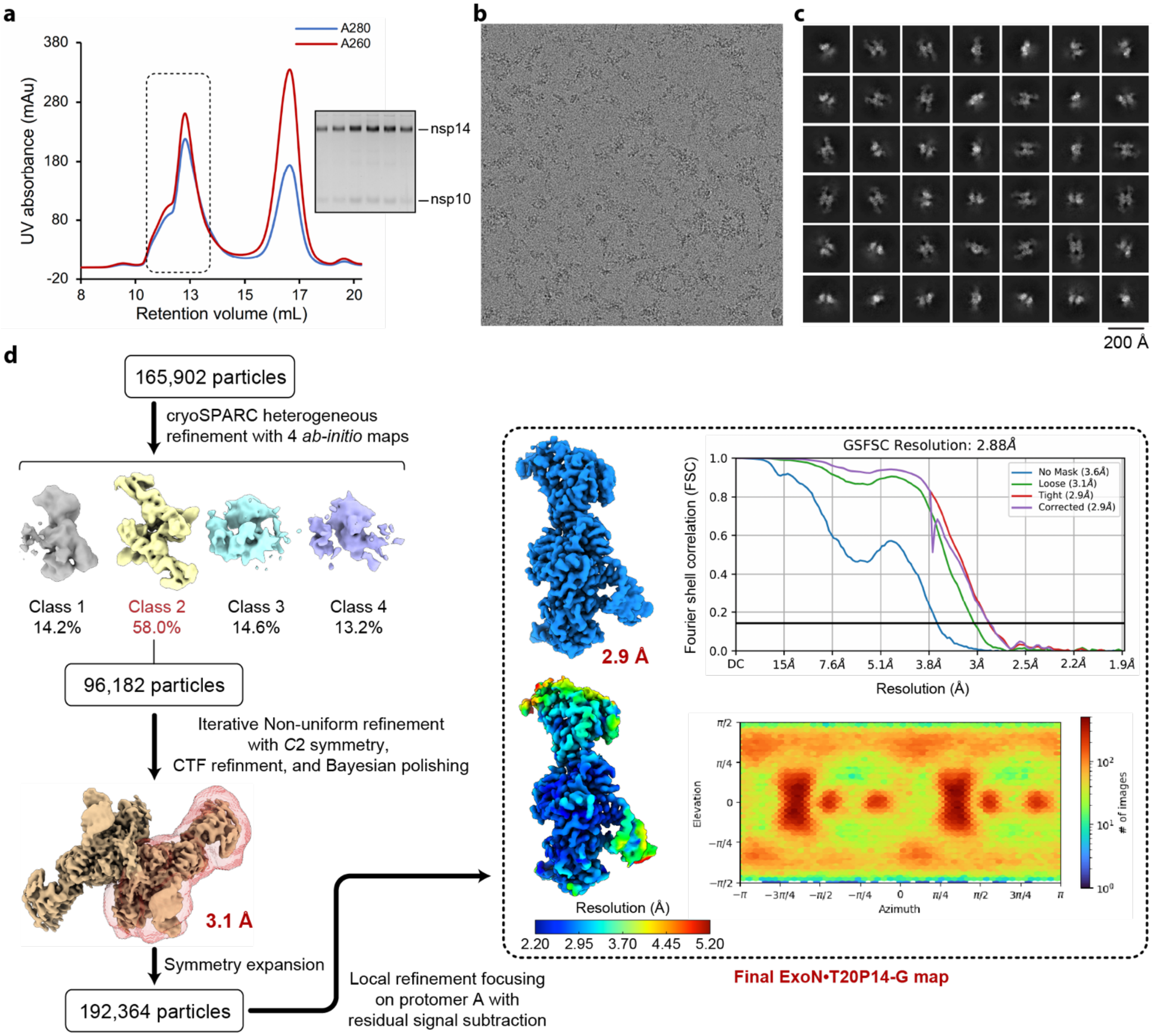

Supplementary Fig. 4: Single-particle cryo-EM analysis of MERS-CoV ExoN•T20P14-G complex. (**a**) SEC purification of MERS-CoV ExoN•T20P14-G complex. The protein compositions in the dotted-line boxed fractions were analyzed by SDS-PAGE and are shown in the embedded gel panel. (**b**) A representative micrograph from the ExoN•T20P14-G complex cryo-EM dataset. (**c**) Representative 2D classes generated from the ExoN•T20P14-G complex cryo-EM dataset. (**d**) Flow chart of cryo-EM image processing and map reconstruction for the ExoN•T20P14-G complex. Heterogeneous refinement with four *ab-initio* reconstructed maps in cryoSPARC generated four major 3D classes. The particles from the predominant 3D class, which shows the structural features corresponding to a dimeric form of ExoN•T20P14-G complex, were selected for iterative non-uniform refinement, CTF refinement, and Bayesian polishing. The final cryo-EM map of the complex, the accompanying half-map FSC plots, local resolution illustrations, and the angular distribution plot of particles used in the final reconstruction are enclosed in the dashed box. Source data are provided as a Source Data file.

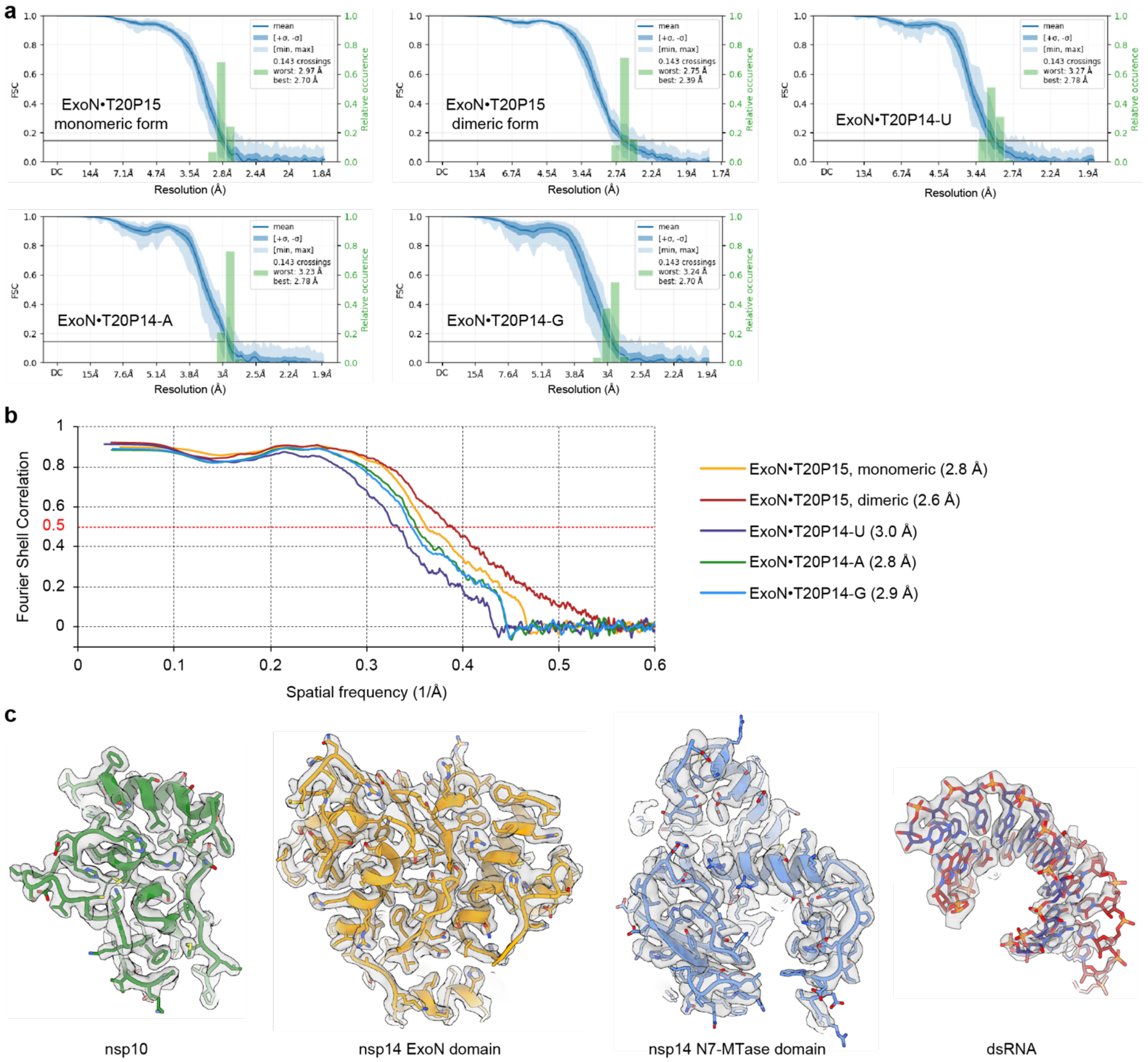

Supplementary Fig. 5: Validation of cryo-EM maps and models. (**a**) Orientation diagnosis and histograms of 3D FSC plots for the raw cryo-EM maps. (**b**) Model-map FSC curves of five atomic structures and their corresponding Resolve density-modified cryo-EM maps from this study were generated from Phenix comprehensive validation results. The model-map resolution for each structure at FSC = 0.5 cutoff is indicated in the figure and summarized in Supplementary Table 1. (**c**) Cryo-EM densities of the DeepEMhancer-processed map superimposed on a structural model of representative regions of MERS-CoV ExoN•RNA complexes determined in this study. Cryo-EM densities are contoured at 4σ and shown as gray surfaces. Source data are provided as a Source Data file.

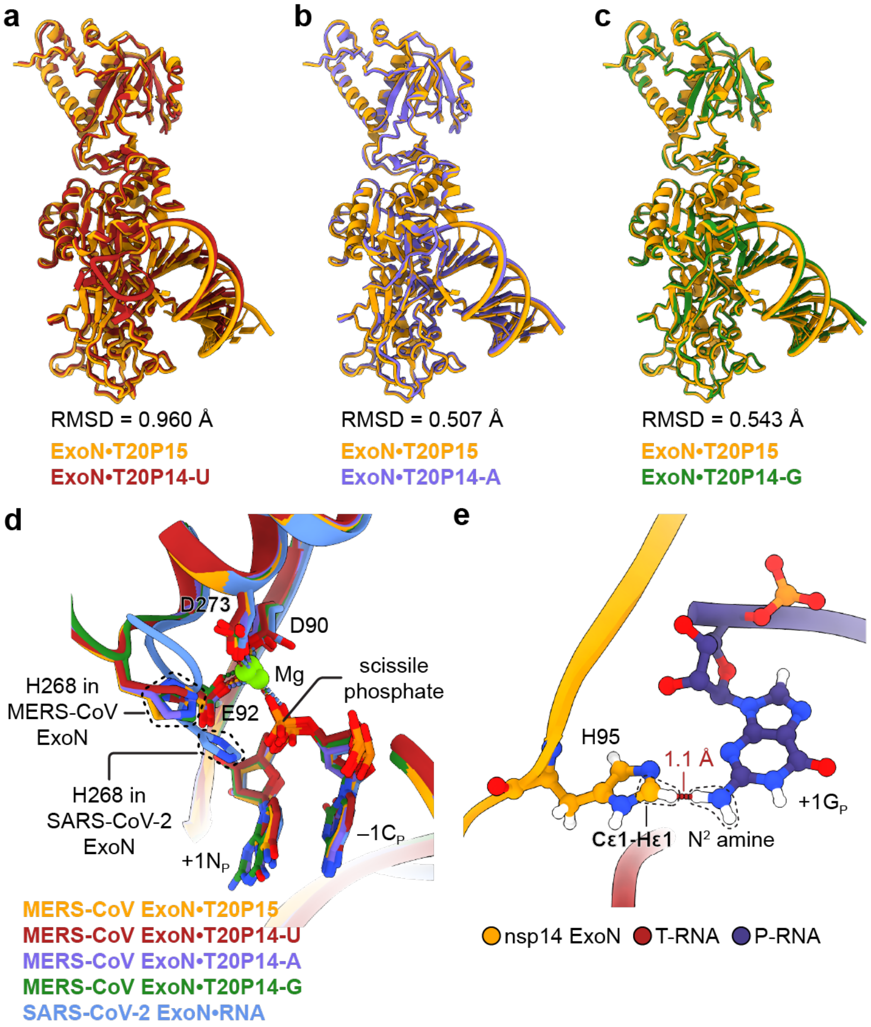

Supplementary Fig. 6: Structural comparison of different MERS-CoV ExoN•RNA complexes. (**a**) Superimposition of MERS-CoV ExoN•T20P15 complex (colored in orange) and the MERS-CoV ExoN•T20P14-U complex (colored in red). (**b**) Superimposition of MERS-CoV ExoN•T20P15 complex (colored in orange) and the MERS-CoV ExoN•T20P14-A complex (colored in medium purple). (**c**) Superimposition of MERS-CoV ExoN•T20P15 complex (colored in orange) and the MERS-CoV ExoN•T20P14-G complex (colored in green). (**d**) Superimposition of the ExoN active site between the four MERS-CoV ExoN•RNA complexes and SARS-CoV-2 ExoN•RNA complex (PDB ID 7N0D). (**e**) Switching the rotamer conformation of H95 in the ExoN•T20P14-G complex to the conformation observed in the ExoN•T20P15 complex results in a clash between H95 and 3'-end guanosine.

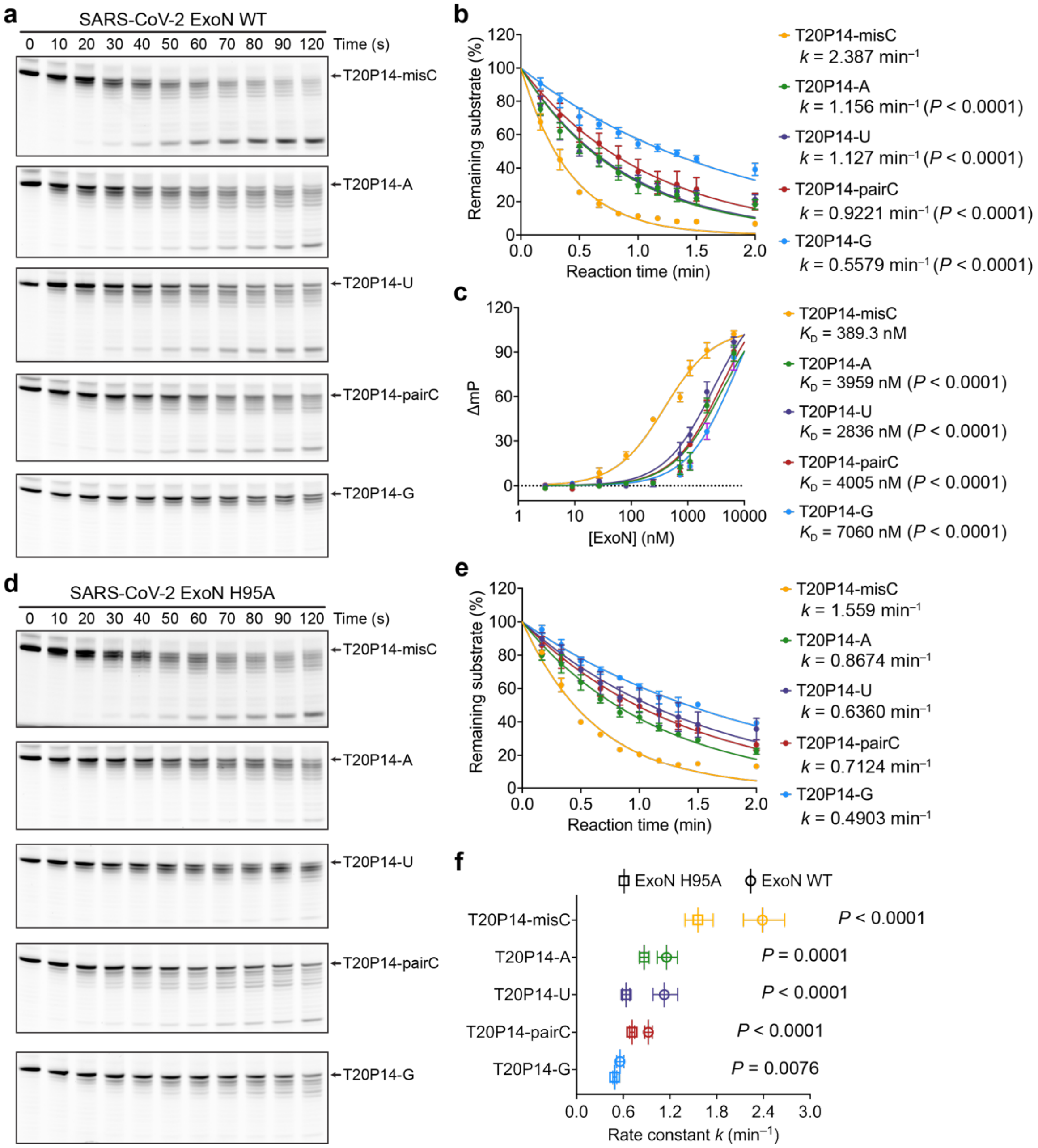

Supplementary Fig. 7: Substrate preference of SARS-CoV-2 ExoN complex. (**a**) Exonucleolytic digestion of RNA substrates bearing different 3'-end nucleotides by SARS-CoV-2 ExoN WT. The reactions were stopped at indicated time points and RNA products were resolved by denaturing PAGE and visualized by FAM imaging. A representative result from three biological replicates is shown. (**b**) Percentages of substrate RNAs remaining shown in (**a**) were quantified using Bio-Rad Image Lab from three independent experiments and are shown as mean ± SEM. The results were plotted in GraphPad Prism using the One-phase decay model. Rate constant (*k*) values are indicated. Statistical analyses were performed using the extra sum-of-squares F test. *P* values for the comparison of rate constants between T20P14-misC and each of the other four RNAs are indicated. (**c**) Fluorescence polarization analysis of the binding between SARS-CoV-2 ExoN complex and different RNA substrates. Each data point represents the mean of six biological

replicates ± SEM. Dissociation constant (K_D) values are indicated. Statistical analyses were performed using the extra sum-of-squares F test. *P* values for the comparison of K_D between T20P14-misC and each of the other four RNAs are indicated. (**d**) Exonucleolytic digestion of RNA substrates bearing different 3'-end nucleotides by SARS-CoV-2 ExoN H95A mutant. A representative result from three biological replicates is shown. (**e**) Percentages of substrate RNAs remaining shown in (**d**) were quantified using Bio-Rad Image Lab from three independent experiments and are shown as mean ± SEM. The results were plotted in GraphPad Prism using the One-phase decay model. Rate constant (*k*) values are indicated. (**f**) Comparison of the rate constants of RNA digestion by SARS-CoV-2 ExoN WT and H95A mutant. Data are shown as best-fit rate constant values ± 95% confidence interval (CI) of three biological replicates determined from curve fitting in (**b**) and (**e**). Statistical analyses were performed using the extra sum-of-squares F test. *P* values are indicated. Source data are provided as a Source Data file.

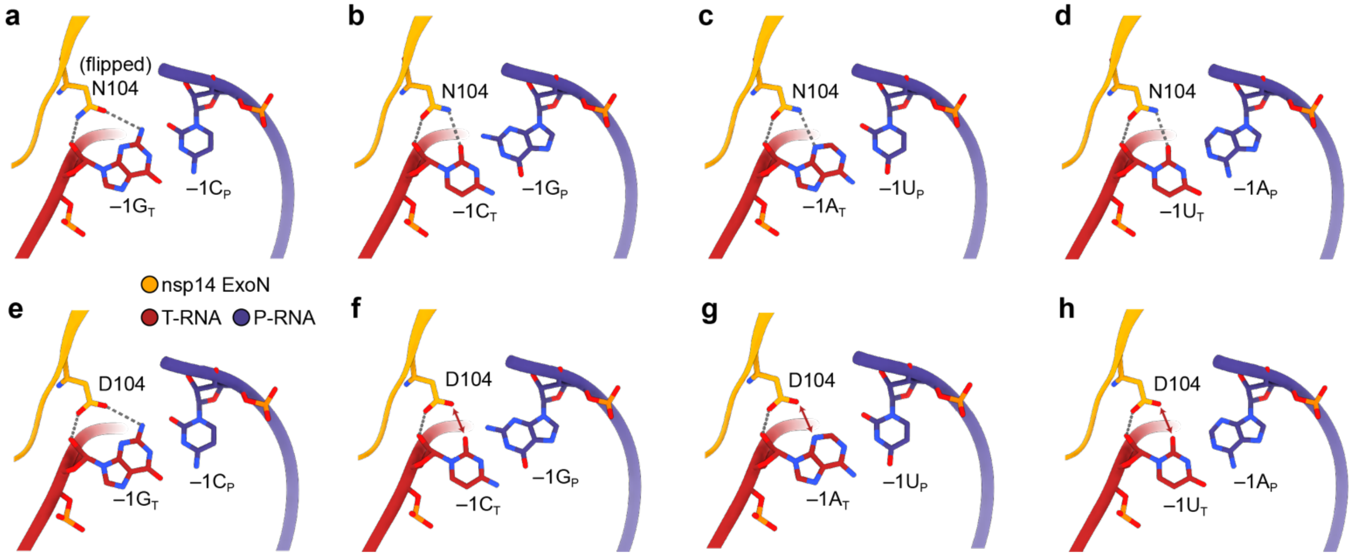

Supplementary Fig. 8: Nsp14 N104 accommodates different base pairs at –1 position of dsRNA substrates. (**a**) Modeled interactions between nsp14 N104 in a side chain-flipped conformation and –1G$_T$. Hydrogen bonds are shown as gray dashed lines. (**b**) Modeled interactions between nsp14 N104 and a C$_T$:G$_P$ base pair at the –1 position. (**c**) Modeled interactions between nsp14 N104 and an A$_T$:U$_P$ base pair at the –1 position. (**d**) Modeled interactions between nsp14 N104 and a U$_T$:A$_P$ base pair at the –1 position. (**e**) Modeled Interactions between nsp14 N104D mutant and a G$_T$:C$_P$ base pair at the –1 position. (**f**) Modeled Interactions between nsp14 N104D mutant and a C$_T$:G$_P$ base pair at the –1 position. The potential repulsion between the D104 carboxylate side chain and the cytosine base of –1C$_T$ is indicated by a red double-headed arrow. (**g**) Modeled Interactions between nsp14 N104D mutant and an A$_T$:U$_P$ base pair at the –1 position. (**h**) Modeled Interactions between nsp14 N104D mutant and a U$_T$:A$_P$ base pair at the –1 position. Source data are provided as a Source Data file.

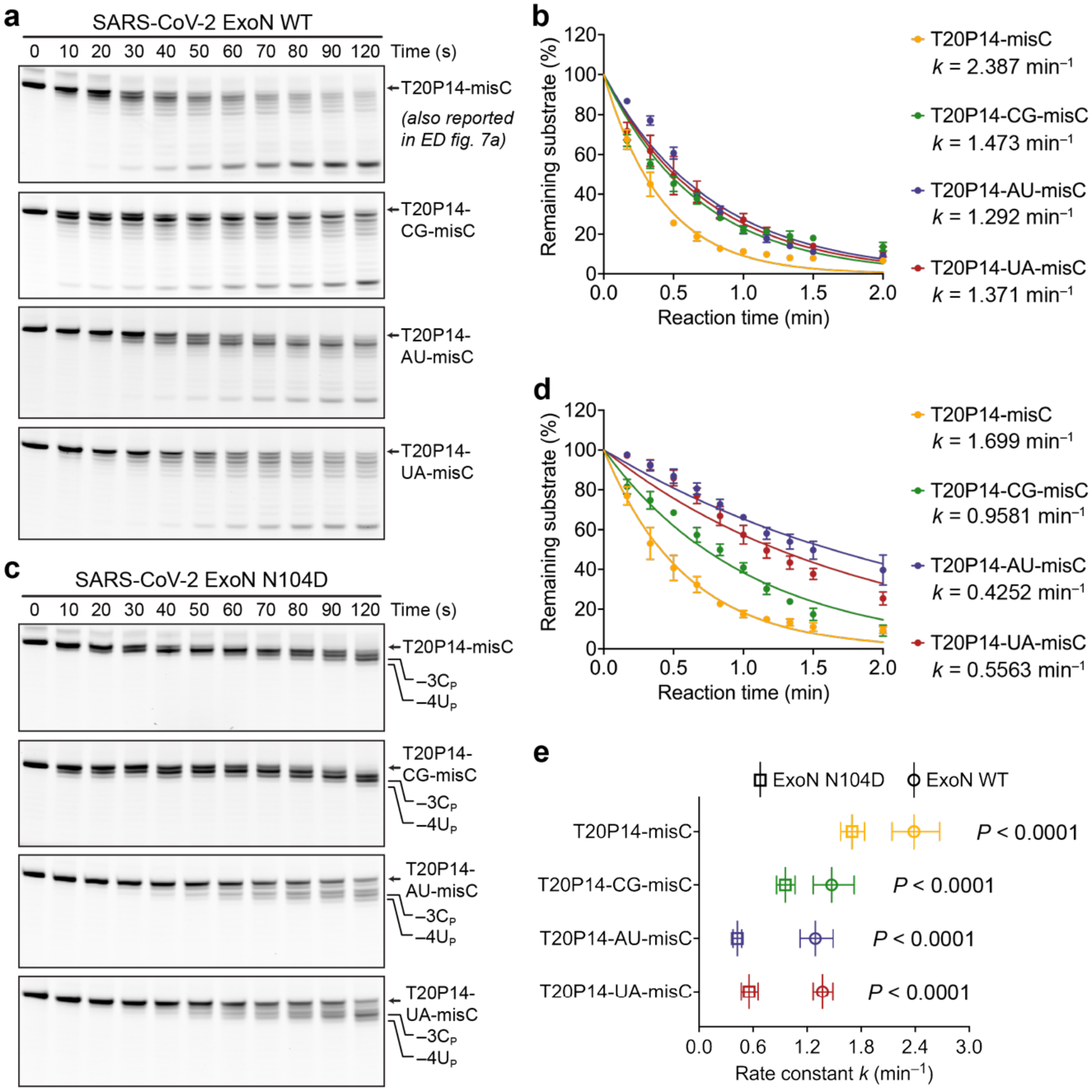

Supplementary Fig. 9: Nsp14 N104 facilitates the sequence-independent digestion of dsRNA substrates by SARS-CoV-2 ExoN. (**a**) Exonucleolytic digestion of RNA substrates bearing different base pairs at the –1 position by SARS-CoV-2 ExoN WT. The reactions were stopped at indicated time points and RNA products were resolved by denaturing PAGE and visualized by FAM imaging. A representative result from three biological replicates is shown. (**b**) Percentages of substrate RNAs remaining shown in (**a**) were quantified using Bio-Rad Image Lab from three independent experiments and are shown as mean ± SEM. The results were plotted in GraphPad Prism using the One-phase decay model. Rate constant (*k*) values are indicated. (**c**) Exonucleolytic digestion of RNA substrates bearing different base pairs at the –1 position by SARS-CoV-2 ExoN N104D mutant. A representative result from three biological replicates is shown. (**d**) Percentages of substrate RNAs remaining shown in (**c**) were quantified using Bio-Rad Image Lab from three independent experiments and are shown as mean ± SEM. The results were plotted in GraphPad Prism using the One-phase decay model. Rate constant (*k*) values are indicated. (**e**) Comparison of the rate constants of RNA digestion by SARS-CoV-2 ExoN WT and N104D mutant. Data are shown as best-

fit rate constant values ± 95% confidence interval (CI) of three biological replicates determined from curve fitting in (**b**) and (**d**). Statistical analyses were performed using the extra sum-of-squares F test. *P* values are indicated. Source data are provided as a Source Data file.

Supplementary Table 1: Cryo-EM data collection, refinement, and validation statistics.

	MERS-CoV ExoN•T20P15, monomeric form (EMDB-72775) (PDB 9YCK)	MERS-CoV ExoN•T20P15, dimeric form (EMDB-72776) (PDB 9YCL)	MERS-CoV ExoN•T20P14-U (EMDB-72777) (PDB 9YCM)	MERS-CoV ExoN•T20P14-A (EMDB-72778) (PDB 9YCN)	MERS-CoV ExoN•T20P14-G (EMDB-72779) (PDB 9YCO)
**Data collection and processing**					
Magnification	130,000	130,000	130,000	130,000	130,000
Voltage (kV)	300	300	300	300	300
Detector	Gatan K3	Gatan K3	Falcon 4i	Falcon 4i	Falcon 4i
Electron exposure ($e^{-}/Å^2$)	51.59	51.59	43.03	43.03	43.03
Defocus range (μm)	1.0–2.0	1.0–2.0	1.0–2.0	1.0–2.0	1.0–2.0
Pixel size (Å)	0.653	0.653	0.9353	0.9353	0.9353
Symmetry imposed	$C1$	$C1$	$C2$	$C1$	$C1$
Initial particle images (no.)	458,363	458,363	107,800	342,866	165,902
Final particle images (no.)	141,301	84,344	57,504	302,690	192,364
Map resolution (Å)	2.8	2.5	2.9	2.9	2.9
FSC threshold	0.143	0.143	0.143	0.143	0.143
Map resolution range (Å)	2.2–5.2	2.2–5.2	2.2–5.2	2.2–5.2	2.2–5.2
**Refinement**					
Initial model used (PDB code)	7N0C	7N0C	7N0C	7N0C	7N0C
Model resolution (Å)	2.8	2.6	3.0	2.8	2.9
FSC threshold	0.5	0.5	0.5	0.5	0.5
Model resolution range (Å)	23.0–2.1	29.1–1.8	36.7–2.3	28.7–2.2	27.7–2.2
Map sharpening B factor ($Å^2$)	97.0	61.5	98.1	82.3	90.6
Model composition					
Non-hydrogen atoms	5663	11284	11380	5646	5647
Protein residues	636	1272	1272	636	636
Nucleotides	32	62	66	31	31
Ligands	6	12	12	6	6
B factors ($Å^2$)					
Protein	68.17	81.36	92.23	76.22	82.41
Nucleic acid	79.28	88.58	117.82	106.61	112.75
Ligand	107.62	109.89	141.94	106.49	118.95
R.m.s. deviations					
Bond lengths (Å)	0.005	0.005	0.004	0.005	0.005
Bond angles (°)	1.012	0.753	0.942	0.733	1.023
Validation					
MolProbity score	1.30	1.28	1.16	1.27	1.36
Clashscore	4.08	4.13	2.26	3.71	4.83
Poor rotamers (%)	0.00	0.00	0.00	0.00	0.00
Ramachandran plot					
Favored (%)	97.46	97.62	97.14	97.46	97.46
Allowed (%)	2.54	2.38	2.86	2.54	2.54
Disallowed (%)	0.00	0.00	0.00	0.00	0.00

Supplementary Table 2: RNA constructs used in this study

Name	Sequence (5′ to 3′)	Modifications	Source	Application
T20P15	GGGAAUGAUUAGGCUAAUUAUUCGUA AUUAGCCUAAUCC	5′-triphosphate	*In vitro* transcription	Exoribonuclease assay (Figs. 1c and 2f); Cryo-EM analysis
T20P14-U	GGGAAAGGGAUUUUAAUAGCUUCGGC UAUUAAAAUCCCU	5′-OH	IDT	Cryo-EM analysis
T20P14-A	GGGAAUGGGAUUUUAAUAGCUUCGGC UAUUAAAAUCCCA	5′-OH	IDT	Cryo-EM analysis
T20P14-G	GGGAACGGGAUUUUAAUAGCUUCGGC UAUUAAAAUCCCG	5′-OH	IDT	Cryo-EM analysis
FAM-T20P14-misC	AGGAAUGGGAUUUUAAUAGCUUCGGC UAUUAAAAUCCCC	5′ 6-FAM	IDT	Exoribonuclease assay (Figs. 2c,d, 4a,d, and 5c,e, Supplementary Figs. 7a,d and 9a,c); Fluorescence polarization assay (Fig. 4c, Supplementary Fig. 7c)
FAM-T20P14-U	AGGACAGGGAUUUUAAUAGCUUCGGC UAUUAAAAUCCCU	5′ 6-FAM	IDT	Exoribonuclease assay (Fig. 4a,d, Supplementary Fig. 7a,d); Fluorescence polarization assay (Fig. 4c, Supplementary Fig. 7c)
FAM-T20P14-A	AGGAAUGGGAUUUUAAUAGCUUCGGC UAUUAAAAUCCCA	5′ 6-FAM	IDT	Exoribonuclease assay (Fig. 4a,d, Supplementary Fig. 7a,d); Fluorescence polarization assay (Fig. 4c, Supplementary Fig. 7c)
FAM-T20P14-pairC	AGGACGGGAAUUUUAAUAGCUUCGGC UAUUAAAAUUCCC	5′ 6-FAM	IDT	Exoribonuclease assay (Fig. 4a,d, Supplementary Fig. 7a,d); Fluorescence polarization assay (Fig. 4c, Supplementary Fig. 7c)
FAM-T20P14-G	AGGAACGGGAUUUUAAUAGCUUCGGC UAUUAAAAUCCCG	5′ 6-FAM	IDT	Exoribonuclease assay (Fig. 4a,d, Supplementary Fig. 7a,d); Fluorescence polarization assay (Fig. 4c, Supplementary Fig. 7c)
FAM-T20P14-CG-misC	AGGAAUCGGAUUUUAAUAGCUUCGGC UAUUAAAAUCCGC	5′ 6-FAM	IDT	Exoribonuclease assay (Fig. 5c,e, Supplementary Fig. 9a,c)

FAM-T20P14-AU-misC	AGGAAUAGGAUUUUAAUAGCUUCGGCUAUUAAAAUCCUC	5′ 6-FAM	IDT	Exoribonuclease assay (Fig. 5c,e, Supplementary Fig. 9a,c)
FAM-T20P14-UA-misC	AGGAAUUGGAUUUUAAUAGCUUCGGCUAUUAAAAUCCAC	5′ 6-FAM	IDT	Exoribonuclease assay (Fig. 5c,e, Supplementary Fig. 9a,c)
