# Supplementary material for: Structural and catalytic diversity of coronavirus proofreading exoribonuclease
Source: Nat Commun. 2025 Dec 8;17:452. doi: 10.1038/s41467-025-67140-6 (PMC12800083; doi:10.1038/s41467-025-67140-6)
Supplement: Supplementary file 2 — Reporting Summary [file 41467_2025_67140_MOESM2_ESM.pdf]

nature portfolio

Corresponding author(s): Yang Yang, Chang Liu

Last updated by author(s): Nov 9, 2025

Reporting Summary

Nature Portfolio wishes to improve the reproducibility of the work that we publish. This form provides structure for consistency and transparency in reporting. For further information on Nature Portfolio policies, see our Editorial Policies and the Editorial Policy Checklist.

Statistics

For all statistical analyses, confirm that the following items are present in the figure legend, table legend, main text, or Methods section.

n/a	Confirmed	
☐	☒	The exact sample size (*n*) for each experimental group/condition, given as a discrete number and unit of measurement
☐	☒	A statement on whether measurements were taken from distinct samples or whether the same sample was measured repeatedly
☐	☒	The statistical test(s) used AND whether they are one- or two-sided<br>*Only common tests should be described solely by name; describe more complex techniques in the Methods section.*
☒	☐	A description of all covariates tested
☒	☐	A description of any assumptions or corrections, such as tests of normality and adjustment for multiple comparisons
☐	☒	A full description of the statistical parameters including central tendency (e.g. means) or other basic estimates (e.g. regression coefficient) AND variation (e.g. standard deviation) or associated estimates of uncertainty (e.g. confidence intervals)
☐	☒	For null hypothesis testing, the test statistic (e.g. *F*, *t*, *r*) with confidence intervals, effect sizes, degrees of freedom and *P* value noted<br>*Give P values as exact values whenever suitable.*
☒	☐	For Bayesian analysis, information on the choice of priors and Markov chain Monte Carlo settings
☒	☐	For hierarchical and complex designs, identification of the appropriate level for tests and full reporting of outcomes
☒	☐	Estimates of effect sizes (e.g. Cohen's *d*, Pearson's *r*), indicating how they were calculated

Our web collection on statistics for biologists contains articles on many of the points above.

Software and code

Policy information about availability of computer code

Data collection	EPU version 3.5
Data analysis	RELION-5.0; MotionCor2; CTFFIND-4.1.13; cryoSPARC v4; Topaz 0.2.5; deepEMhancer 0.14; UCSF pyem v0.5; ISOLDE v1.7; Phenix-1.20; Coot-0.9.8; UCSF ChimeraX 1.7; MolProbity included in Phenix-1.20; Espript 3.0 web server, GraphPad Prism 10.

For manuscripts utilizing custom algorithms or software that are central to the research but not yet described in published literature, software must be made available to editors and reviewers. We strongly encourage code deposition in a community repository (e.g. GitHub). See the Nature Portfolio guidelines for submitting code & software for further information.

Data

Policy information about availability of data

All manuscripts must include a data availability statement. This statement should provide the following information, where applicable:

- Accession codes, unique identifiers, or web links for publicly available datasets
- A description of any restrictions on data availability
- For clinical datasets or third party data, please ensure that the statement adheres to our policy

Atomic coordinates of five structures determined in this study have been deposited in the Protein Data Bank with accession codes 9YCK [https://www.rcsb.org/structure/9YCK] (MERS-CoV ExoN•T20P15 complex, monomeric form), 9YCL [https://www.rcsb.org/structure/9YCL] (MERS-CoV ExoN•T20P15 complex, dimeric form), 9YCM [https://www.rcsb.org/structure/9YCM] (MERS-CoV ExoN•T20P14-U complex), 9YCN [https://www.rcsb.org/structure/9YCN] (MERS-CoV

ExoN•T20P14-A complex), and 9YCO [https://www.rcsb.org/structure/9YCO] (MERS-CoV ExoN•T20P14-G complex). The cryo-EM maps have been deposited in the Electron Microscopy Data Bank with accession numbers EMD-72775 [https://www.ebi.ac.uk/emdb/EMD-72775] (MERS-CoV ExoN•T20P15 complex, monomeric form), EMD-72776 [https://www.ebi.ac.uk/emdb/EMD-72776] (MERS-CoV ExoN•T20P15 complex, dimeric form), EMD-72777 [https://www.ebi.ac.uk/emdb/EMD-72777] (MERS-CoV ExoN•T20P14-U complex), EMD-72778 [https://www.ebi.ac.uk/emdb/EMD-72778] (MERS-CoV ExoN•T20P14-A complex), and EMD-72779 [https://www.ebi.ac.uk/emdb/EMD-72779] (MERS-CoV ExoN•T20P14-G complex). Source data are provided with this paper.

Research involving human participants, their data, or biological material

Policy information about studies with human participants or human data. See also policy information about sex, gender (identity/presentation), and sexual orientation and race, ethnicity and racism.

Reporting on sex and gender	N/A
Reporting on race, ethnicity, or other socially relevant groupings	N/A
Population characteristics	N/A
Recruitment	N/A
Ethics oversight	N/A

Note that full information on the approval of the study protocol must also be provided in the manuscript.

Field-specific reporting

Please select the one below that is the best fit for your research. If you are not sure, read the appropriate sections before making your selection.

☒ Life sciences ☐ Behavioural & social sciences ☐ Ecological, evolutionary & environmental sciences

For a reference copy of the document with all sections, see nature.com/documents/nr-reporting-summary-flat.pdf

Life sciences study design

All studies must disclose on these points even when the disclosure is negative.

Sample size	No statistical methods were used to predetermine sample size. Sufficient cryo-EM data were collected to achieve adequate map resolutions for model building. The sample size was based on previous studies. For functional assays, the number of times that experiments were repeated is addressed under "Replication" below.
Data exclusions	According to the standard practice in the field, bad cryo-EM images, including those with heavy ice contaminations, with excessive motion, or without clearly visible particles, were excluded from downstream particle picking and map reconstruction.
Replication	All biochemical assays were repeated at least three times. Each replicate was an independent experiment and did not represent re-assay of the same material. All attempts at replication were successful.
Randomization	Randomization was not relevant to our study because our study did not involve the allocation of samples/organisms/participants into experimental groups.
Blinding	Investigators were not blinded to group allocation because group allocation was not involved in our study. Investigators were not blinded during data collection because the data being collected were quantitative in nature (gels or numbers of particles extracted from cryo-EM data) and were not prone to subjective interpretation.

Reporting for specific materials, systems and methods

We require information from authors about some types of materials, experimental systems and methods used in many studies. Here, indicate whether each material, system or method listed is relevant to your study. If you are not sure if a list item applies to your research, read the appropriate section before selecting a response.

Materials & experimental systems

n/a	Involved in the study
☒	☐ Antibodies
☒	☐ Eukaryotic cell lines
☒	☐ Palaeontology and archaeology
☒	☐ Animals and other organisms
☒	☐ Clinical data
☒	☐ Dual use research of concern
☒	☐ Plants

Methods

n/a	Involved in the study
☒	☐ ChIP-seq
☒	☐ Flow cytometry
☒	☐ MRI-based neuroimaging

Plants

Seed stocks	N/A
Novel plant genotypes	N/A
Authentication	N/A
